# Comprehensive Analysis of m7G-Related Genes and Chronic Hepatitis B: Diagnostic Markers, Immune Microenvironment Regulation, Disease Progression

**DOI:** 10.1155/2023/9471520

**Published:** 2023-05-10

**Authors:** Rongzheng Zhang, Yan Xia, Jianming Dong, Xiaomei Ju, Kun Zhou, Xinyang Cao, Jiaqi Li, Jiaqiu Ru, Mengrui Guo, Shuyun Zhang

**Affiliations:** ^1^Scientific Research Center, The Second Affiliated Hospital of Harbin Medical University, Harbin, China; ^2^Department of Clinical Laboratory, Beidahuang Industry Group General Hospital, Harbin, China; ^3^The Second Affiliated Hospital of Xiamen Medical College, Xiamen, China

## Abstract

Chronic hepatitis B (CHB) is a major public health problem in the world. It is the main cause of liver cirrhosis and liver cancer. Although many important roles of RNA modification in stem cells or tumor diseases have been identified, the role of N7-methylguanosine (m7G) modification in the process of chronic HBV infection has not been clearly defined. Therefore, we conducted a systematic analysis on the process of chronic HBV infection. We found that a total of 18 m7G-related genes were altered in chronic HBV infection, and then we screened out CHB potential diagnostic biomarkers using machine learning and random forest methods. RT-qPCR was performed on the samples of healthy people and CHB, which further verified the possibility of being a diagnostic marker. Then, we typed CHB patients based on these 18 genes. We found that the immune microenvironment of different subtypes was different. Among them, patients with subtype-Ⅰ had severe immune response, that is, relatively serious immune cell infiltration, rich immune pathways, relatively many HLA genes, and immune checkpoints. Finally, we conducted an in-depth discussion on our m7G-related genes, and found that m7G gene related to immune cell infiltration may be involved in the disease progression of CHB patients, which was also confirmed in the GSE84044 dataset. In conclusion, m7G-related genes can not only serve as diagnostic markers of CHB, but also participate in the regulation of immune microenvironment and play an important role in the progression of CHB.

## 1. Introduction

Despite the popularization of vaccines and the application of antiviral drugs, there are still more than 250 million patients with chronic hepatitis B (CHB) in the world, some of whom can progress to liver fibrosis, cirrhosis, and hepatocellular carcinoma (HCC), resulting in nearly 1 million deaths every year [1–3]. Studies have reported that HBV infection is the most important risk factor for HCC. HBV-related HCC accounts for more than 80% of all HCC patients in the world [4]. Persistent HBV infection can lead to varying degrees of liver damage, eventually leading to hepatitis, liver fibrosis, cirrhosis, and HCC [5]. The process of chronic HBV infection usually includes different clinical stages, each of which may last for decades [6]. Therefore, it is of great significance to conduct in-depth discussion and research on the chronic stage of HBV infection, to prevent the further progress of patients' diseases and to individualize treatment.

With the rapid development of next generation sequencing, at least 170 different post transcriptional RNA modifications are known, which widely exist in various RNA types of organisms. These modifications range from methylation to complex chemical structures, of which methylation is the most abundant [7, 8]. N7-methylguanosine (m7G) is a common 5′ cap modification of mRNA and an internal modification in various noncoding RNAs, and can improve the stability of mRNA, which is very important for effective gene expression and cell viability [9–12]. m7G modification plays a key role in regulating RNA processing, metabolism, and function [13] in the transcription process of RNA polymerase II, the 5′ end of the new RNA is covalently modified by monomethylated guanosine [14] and m7G cap binding nuclear cap binding complex (CBC) [15]. Mature mRNAs exchange CBC for eIF4E, a rate-limiting translation factor controlled by mTOR [16]. The translation control regulated by mTOR combines immune cell signals with inflammatory response, which is the basis of effective antiviral response [17]. On the other hand, methylation in RNA also plays a role in immune regulation [18]. m7G cap methyltransferase RNMT is induced during T-cell activation and is necessary to support a significant increase in RNA production, processing, and translation of T-cell amplification [19]. Methylation weakens the antigenicity of RNA and inhibits the immune response. After methylation modification, the immunogenicity of RNA is weakened or disappeared, and innate immunity is no longer triggered [20]. As a noncytopathic virus, HBV is widely believed to mediate chronic liver injury through abnormal immune attack. Infiltration of monocytes/macrophages, NK cells, NKT cells, T cells, and regulatory T cells (Treg) can lead to chronic liver inflammation and aggravate the progress of CHB disease [21]. Our previous studies have shown that immune cells can predict different stages of HBV infection [22]. However, the role of m7G in the process of chronic HBV infection and disease progression and its correlation with immune microenvironment are still unknown. Recent studies have shown that a large number of genes are involved in the regulation of m7G modification. We collected 29 m7G-related genes from previous literatures. We systematically evaluated the mechanism of m7G-related genes in patients with CHB. On the one hand, we found that the expression of m7G-related genes was significantly different between CHB patients and healthy subjects, and LARP1 and GEMIN5 could be used as potential biomarkers of CHB. On the other hand, m7G-related genes have a strong correlation with immune cell infiltration, which indicates that m7G is involved in regulating the immune microenvironment of CHB. Subsequently, we found that the change of m7G-related genes was related to the progress of CHB, and predicted that m7G might be used as a predictor of the development of patients with CHB to the severity of the disease.

Our results may contribute to the role of m7G modification in the context of chronic HBV infection. This is of great significance for the treatment of CHB patients in the future and for delaying the development of the disease.

## 2. Materials and Methods

**Figure 1:**
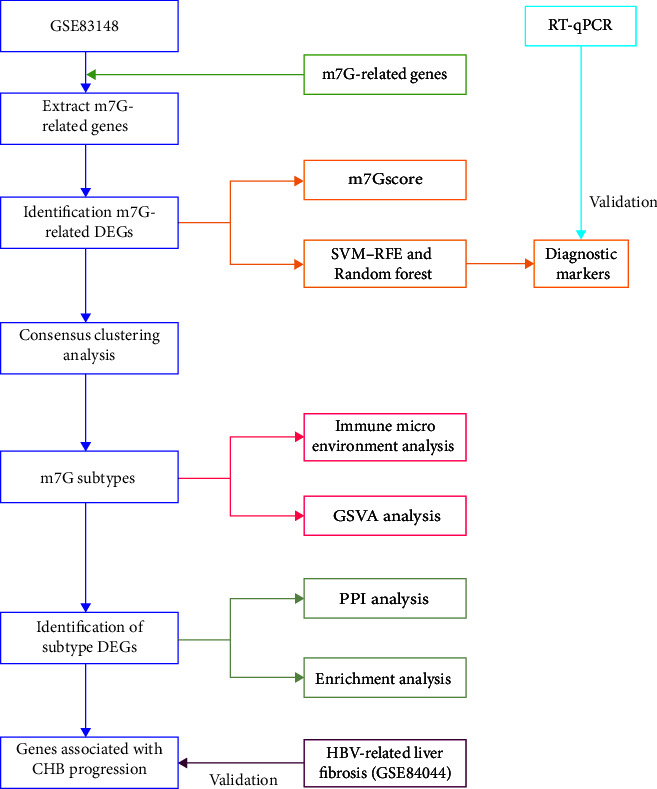
Flowchart of this study.

The procedure in this paper follows a flowchart (Figure 1).

### 2.1. Collection of Clinical Specimens

Thirty healthy people and 39 CHB peripheral blood samples were collected in the Second Hospital of the Harbin Medical University. All healthy people were positive for HBsAb. All CHB patients were positive for HBsAg, and the course of disease lasted for more than 6 months. Both healthy people and CHB patients were older than 18 years and obtained informed consent and permission from the medical ethics committee of the Second Hospital of the Harbin Medical University. Their clinical information is shown in Table S1.

### 2.2. Data Acquisition and Preprocessing

The public datasets GSE83148 and GSE84044 of the same platform file (GPL570) used in this paper were downloaded from Gene Expression Omnibus (GEO) database (https://www.ncbi.nlm.nih.gov/geo/). The GSE83148 dataset contains 128 liver samples, which are divided into two types: CHB and healthy. All liver samples were HBsAg positive or HBV DNA positive. The GSE84044 dataset contains 124 HBV-related liver fibrosis samples. In addition, we transformed the probe set of expression files into gene symbols. If there is no corresponding gene symbol, the probe set will also be deleted by us. Then, the expression value of the probe set corresponding to the same gene symbol was kept at the average value. We obtained 29 m7G-related genes in the previous literature (12). The m7G-related gene loci were visualized by Circos diagram, and the correlations of m7G-related genes in all samples were evaluated by Spearman correlation analysis.

### 2.3. Identification of Differentially Expressed Genes

m7G-related genes were first extracted. Then, the expression differences of m7G-related genes in healthy and CHB samples were compared with R (“limma” package). The heatmap and boxplot of differentially expressed genes (DEGs) were drawn by R (“pheatmap” and “ggpubr” packages).

### 2.4. Construction of m7Gscore

Principal component analysis (PCA) was performed for m7G-related DEGs, and the scores of PC1 and PC2 were calculated by PCA function “prcomp” of R software. The m7Gscore was calculated by the formula: m7Gscore = Ʃ(PC1i + PC2i). In the formula, “*i*” represents m7G-related DEGs.

### 2.5. Screening of Diagnostic Markers

We used two methods to screen for potential diagnostic markers in DEGs. Random forest algorithm is an effective method to obtain the most accurate variable class by constructing decision trees [23]. Support vector machine-recursive feature elimination (SVM-RFE) iteratively deletes the features with the least weight from the rank until all features are excluded, and in each iteration, the current SVM-RFE model is evaluated by *k*-fold cross-validation to obtain the most effective variable [24]. Then, we take the intersection of genes obtained by the two methods and display them using Venn diagram. Finally, the ability of genes to distinguish healthy people from CHB was evaluated by receiver operating characteristic (ROC) curve analysis.

### 2.6. Verification by RT-qPCR Experiment

First, peripheral blood mononuclear cells (PBMCs) were extracted from peripheral blood samples of healthy people and CHB patients with Density Reagent (LTS10770125, TBDscienceHY, Tianjin, China), and then total RNA in PBMCs was extracted by Trizol (SM129-02, Sevenbio, Beijing, China). One microgram of RNA was converted to cDNA using a reverse transcription kit (1119ES60, Yeasen, China). SYBR Green Master Mix kit (11184ES03, Yeasen, China) and RT-qPCR instrument (SLAN-96p Shanghai hongshi, China) were used for RT-qPCR. To ensure the accuracy of the results, all samples were tested three times. The *Δ*CT results were processed with the values of glyceraldehyde-3-phosphate dehydrogenase (GAPDH). The primer sequences are shown in Table S2.

### 2.7. Consensus Clustering Analysis

In order to reveal the biological characteristics of m7G methylation in patients with hepatitis B, the samples were divided into two subtypes according to the m7G-related DEGs with the help of R (“ConsensusClusterPlus” package) consensus clustering algorithm, and PCA was used to analyze the visualization of the subtypes [25, 26]. In addition, the clinical information in the subtypes was plotted as a percentage histogram. Then the expressions of m7G-related DEGs in the two subtypes were compared, and heatmap and boxplot were drawn.

### 2.8. Immune Microenvironment Analysis

In order to find the deep connection between the immune microenvironment of CHB patients and m7G modification, we used single-sample Gene Set Enrichment Analysis (ssGSEA) to represent the relative abundance of immune cells or immune pathways with enrichment fraction, and then quantitatively evaluated the immune microenvironment of CHB samples, and analyzed the infiltration degree of 23 immune cells and 18 immune pathways among two m7G subtypes [27]. In addition, Spearman correlation analysis was used to identify the correlation between m7G DEGs and immune cells. Then R packages were used to compare the DEGs related to checkpoint and antigen processing and presentation pathways in different groups. The immune pathways were derived from IMMPORT database (https://www.immport.org/home), and the immune checkpoint genes were used as the 18th immune pathway.

### 2.9. DEGs Identification among m7G Subtypes and PPI Network Construction

Using |log2 FC| > 1 and adjusted *p* values < 0.05 as filters, we used R (“limma” package) to identify DEGs among the two m7G subtypes. Then, the volcano plot was drawn to visualize the DEG. The STRING database (https://string-db.org/cgi/input.pl) is a database for analyzing and predicting interactions between proteins. And then we go through it, interaction score >0.4 was set as a filter and hidden disconnected nodes in the network to construct protein–protein interaction (PPI) network to evaluate the relationship between DEGs.

### 2.10. Enrichment Analysis

To determine the underlying function and pathway mechanisms of DEGs between different m7G subtypes, we used three different enrichment analyses. The enrichment analysis of Gene Ontology (GO), Kyoto Encyclopedia of Genes and Genomes (KEGG), and Gene Set Variation Analysis (GSVA) was realized by R (“clusterProfiler,” “org.Hs.eg.db,” “enrichplot,” “GSEABase,” “GSVA,” “limma” packages). Among them, GSVA enrichment analysis is run based on c2.cp.kegg.v7.2.symbols.gmt files downloaded by the Molecular Signatures Database (MSigDB, http://www.gsea-msigdb.org/gsea/msigdb/index.jsp). |logFC| > 0.1 and adjusted *p* value < 0.05 were used as the filtering conditions of the analysis method, and the filtering results were considered to be significant.

### 2.11. Clinical Information Analysis

After consensus clustering analysis, alanine aminotransferase (ALT) and aspartate aminotransferase (AST) in clinical information were selected as supplementary data. Patients in the dataset were divided into ALT > 40 and ≤40 groups and AST > 35 and ≤35 groups. Then we plotted the percentages of ALT and AST in the m7G subtypes into histogram. In addition, m7Gscore of different ALT and AST subgroups were compared.

### 2.12. Identification of m7G-Related Genes Associated with CHB Progression

We predicted the genes related to the progression of CHB through the correlation between m7G DEGs and immune cells, and verified our hypothesis with GSE84044 dataset. The boxplot is drawn with R (“ggpubr” package).

### 2.13. Statistical Analysis

All statistical analyses in this paper are calculated by R software (version 4.1.2). Spearman correlation analysis was used to evaluate the correlation of m7G-related genes in samples. Wilcoxon test was used to compare the differences between the two groups. The results of PCR did not conform to normal distribution, so Wilcoxon test was also used. ROC curve was used to judge the diagnostic efficacy of genes. Spearman correlation analysis was used to calculate the correlation between m7G-related DEGs and immune cell infiltration or immune activity pathways. In order to evaluate the clinical characteristics between m7G subtypes, *χ*^2^ test was used. *p* < 0.05 was considered statistically significant.

## 3. Results

### 3.1. Correlation and Differential Expression Analysis of m7G-Related Genes and Construction of m7Gscore System

**Figure 2:**
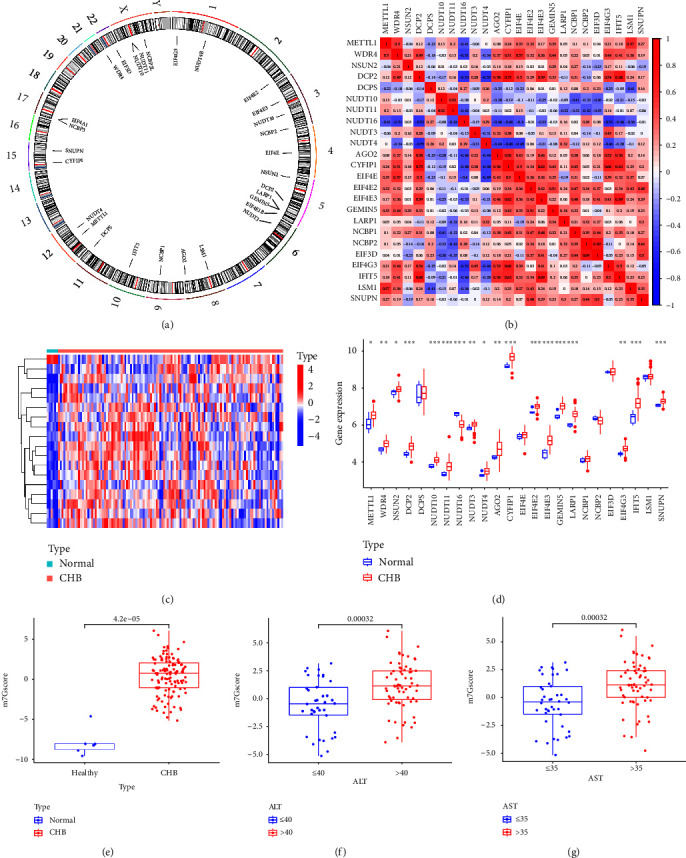
Identification of m7G-related DEGs. (a) Circle diagram of 31 m7G-related gene locations. (b) Correlation analysis of m7G-related genes in total samples. (c) The heatmap of m7G-related DEGs in CHB samples and normal samples. (d) The boxplot of m7G-related DEGs in CHB samples and normal samples. (e) The boxplot of m7G scores in normal and CHB patients. (f) The boxplot of m7G scores in CHB patients with normal and elevated ALT. (g) The boxplot of m7G scores in CHB patients with normal and elevated AST. DEGs, differentially expressed genes; CHB, chronic hepatitis B; ALT, alanine aminotransferase; AST, spartate aminotransferase.  ^*∗*^*p* < 0.05,  ^*∗∗*^*p* < 0.01,  ^*∗∗∗*^*p* < 0.001.

A total of 29 m7G-related genes were included in this paper. In order to understand them more clearly, we used the circle diagram to visualize the gene location (Figure 2(a)). Next, we analyzed the correlation between m7G-related genes in the overall sample, and found that there was a close correlation between m7G-related genes (Figure 2(b)). Then, we analyzed the expression of m7G-related genes in CHB and normal samples. Results as shown in Figures 2(c) and 2(d), there were differences in the expression levels of 18 m7G-related genes between the two groups, of which 17 genes were upregulated and one gene was downregulated, indicating that m7G modification may participate in the regulatory process during chronic HBV infection. Then, based on the m7G DEGs, we constructed a related scoring system by PCA analysis, and named it m7Gscore. Then we found that the m7Gscore of CHB patients was significantly higher than that of normal people (Figure 2(e)). In addition, the m7Gscore in the ALT > 40 and AST > 35 groups was higher than that in the corresponding normal group (*p* < 0.001) (Figures 2(f) and 2(g)), suggesting that m7Gscore may be positively correlated with the degree of CHB inflammatory response.

### 3.2. Identify and Validate the Diagnostic Ability of m7G-Related DEGs

**Figure 3:**
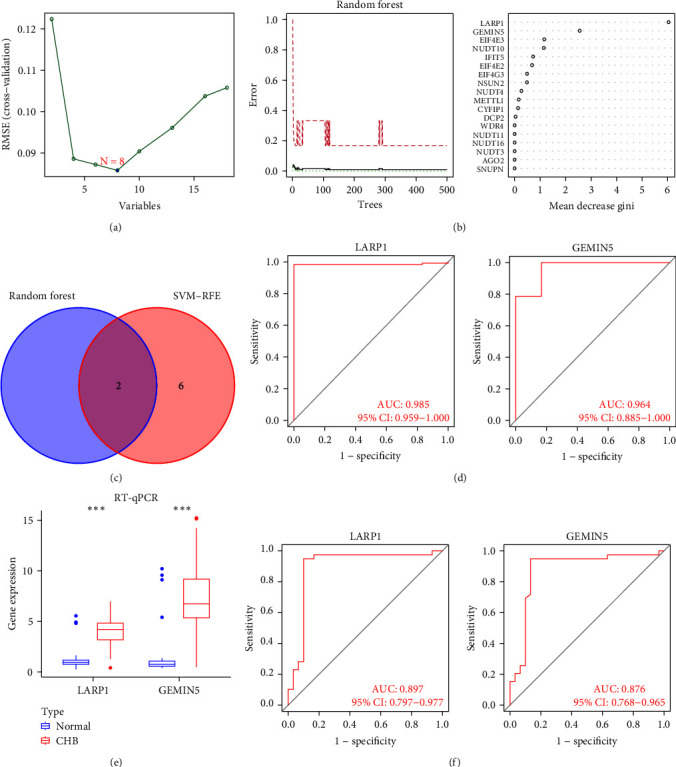
Screening of diagnostic markers for CHB patients. (a) The SVM-RFE algorithm identified eight genes. (b) The random forest algorithm identified two genes. (c) The random forest algorithm and machine learning algorithm overlap diagnostic markers. (d) The ROC curves of LARP1 and GEMIN5. (e) The boxplot of LARP1 and GEMIN5 expression levels measured by RT-PCR in peripheral blood samples from normal people (*n* = 30) and CHB patients (*n* = 39). (f) The ROC curves of LARP1 and GEMIN5 in RT-qPCR results. CHB, chronic hepatitis B; SVM-RFE, support vector machine-recursive feature elimination; ROC, receiver operating characteristic; RT-qPCR, real-time quantitative PCR.  ^*∗*^*p* < 0.05,  ^*∗∗*^*p* < 0.01,  ^*∗∗∗*^*p* < 0.001.

We used SVM-RFE methods to obtain eight genes that can be used as diagnostic markers (Figure 3(a)). Then, we use the random forest method to obtain the genes whose importance score is greater than 2 (Figure 3(b)). For the accuracy of the biomarkers, we selected the overlapping genes (*LARP1* and *GEMIN5*) of the two algorithms (Figure 3(c)). We found that *LARP1* (AUC = 0.985) and *GEMIN5* (AUC = 0.964) have high-diagnostic value (Figure 3(d)). In order to determine whether *LARP1* and *GEMIN5* can be used as diagnostic markers, we performed validation by RT-qPCR. First, we found that *LARP1* and *GEMIN5* were significantly upregulated in patients with CHB (*p* < 0.001) (Figure 3(e)). Next, we confirmed the diagnostic ability of *LARP1* (AUC = 0.897) and *GEMIN5* (AUC = 0.876) by ROC curve (Figure 3(f)).

### 3.3. Consensus Clustering Analysis of m7G-Related DEGs

**Figure 4:**
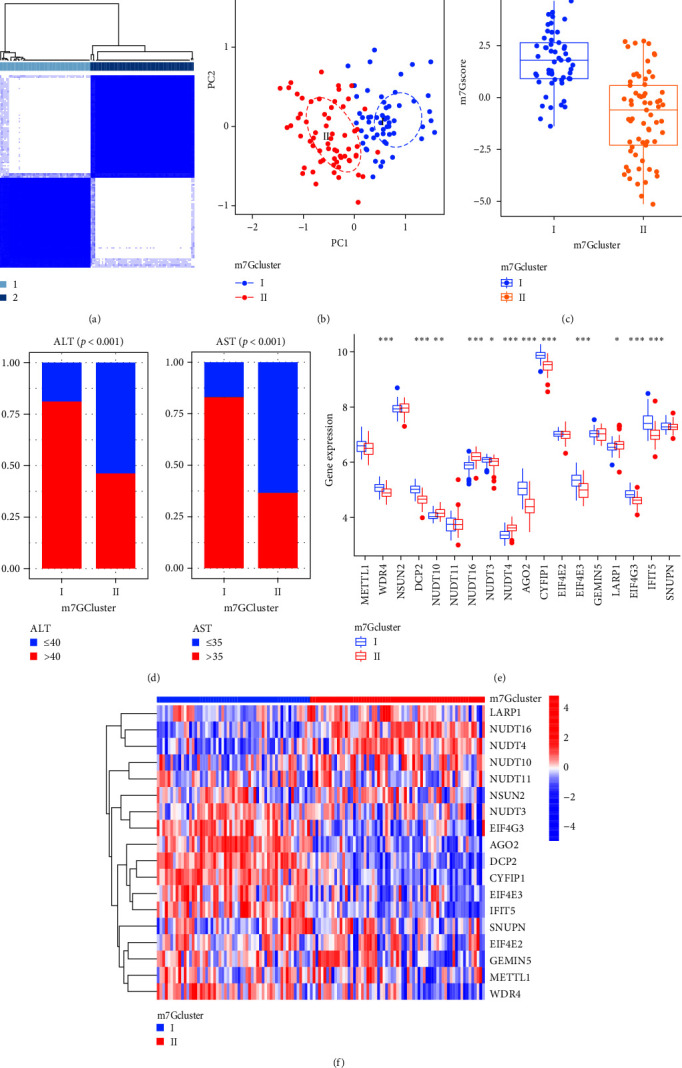
Identification of m7G modified subtypes (a) Consensus clustering of the m7G-related DEGs for *k* = 2. (b) PCA showed that there was a significant separation between the two m7G subtypes. (c) The boxplot of m7G scores for two subtypes. (d) The distribution of ALT and AST information in patients with two m7G subtypes. (e) The boxplot of m7G DEGs in two subtypes. (f) The heatmap of m7G DEGs in two subtypes. DEGs, differentially expressed genes; PCA, principal component analysis; ALT, alanine aminotransferase; AST, aspartate aminotransferase.  ^*∗*^*p* < 0.05,  ^*∗∗*^*p* < 0.01,  ^*∗∗∗*^*p* < 0.001.

In order to study the relationship between m7G modification and CHB, we first performed consensus clustering analysis on CHB samples based on m7G-related DEGs. According to Figure S1(a)–S1(c), it can be seen that the stability of CHB samples is the highest when divided into two subtypes. Therefore, we selected two subtypes with different m7G modifications for research (Figures 4(a) and 4(b)). Among them, subtype-Ⅰ includes 57 CHB patients, and subtype-Ⅱ includes 65 CHB patients. Further analyzing the relationship between the two subtypes and m7Gscore, we found that m7Gscore of subtype-Ⅰ was higher than that of subtype-Ⅱ (*p* < 0.001) (Figure 4(c)). In addition, ALT > 40 and AST > 35 subtype-Ⅰ was significantly more than subtype-Ⅱ, suggesting that the liver inflammation in patients with subtype-Ⅰ was the most severe (Figure 4(d)). Next, we observed that the expression levels of 12 m7G-related DEGs were different among the two subtypes (Figures 4(e) and 4(f)). It is suggested that different genes involved in m7G modification play different roles in liver injury.

### 3.4. Immune Landscape of Two m7G Subtypes

**Figure 5:**
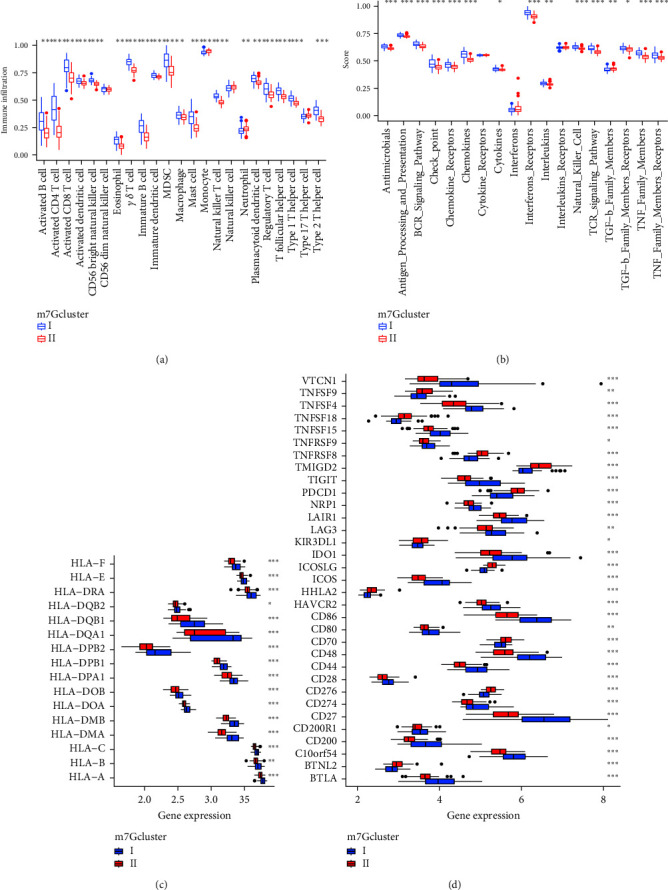
Analysis of immune microenvironment of m7G subtype. (a) The boxplot of immune cell infiltration of two subtypes. (b) The boxplot of immune pathway scores of two subtypes. (c) The boxplot of HLA gene expression of two subtypes. (d) The boxplot of immune checkpoint related gene expression of two subtypes. HLA, human leukocyte antigen.  ^*∗*^*p* < 0.05,  ^*∗∗*^*p* < 0.01,  ^*∗∗∗*^*p* < 0.001.

Chronic HBV infection is a complex process involving the interaction between host immune system and virus. Next, we analyzed the differences of immune microenvironment among different m7G subtypes. First, we used ssGSEA to analyze the immune cell infiltration and pathways. We found that the immune cells and pathways of the two subtypes were different. The immune cell infiltration level and pathway of subtype-Ⅰ were generally higher than that of subtype-Ⅱ (Figures 5(a) and 5(b)). As a key factor of antigen presentation, HLA gene also showed a relatively high level in subtype-Ⅰ with the heaviest inflammatory response (Figure 5(c)), and the immune checkpoints involved in immunosuppression were also most expressed in subtype-Ⅰ (Figure 5(d)). The above results suggest that subtype-Ⅰ has a higher inflammatory response, the degree of immune injury is relatively severe, and the relatively more inflammatory response further increases the possibility of developing cirrhosis.

### 3.5. Identification of DEGs among m7G Subtypes and Enrichment Analysis

**Figure 6:**
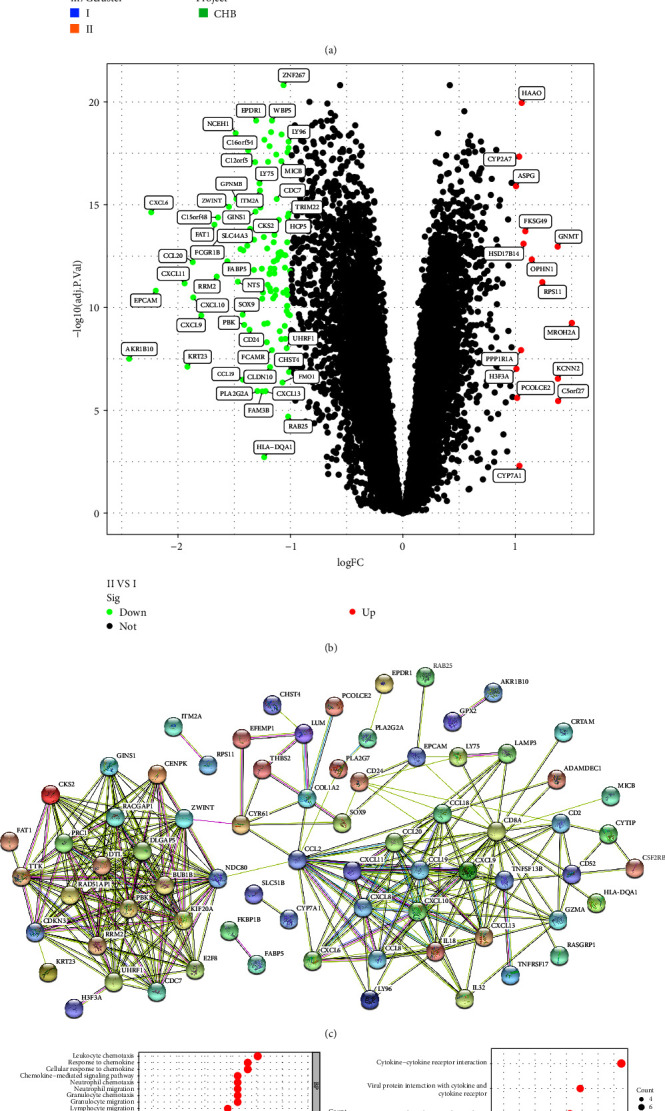
Identification of DEGs of m7G subtypes and PPI analysis and enrichment analysis. (a) The heatmap of biological pathways between subtype-Ⅰ and subtype-Ⅱ. (b) The volcano plot of DEGs of two m7G subtypes. (c) The PPI analysis of DEGs of m7G subtypes. (d) The GO enrichment analysis of DEGs of m7G subtypes. (e) The KEGG pathway analysis of DEGs of m7G subtypes. DEGs, differentially expressed genes; PPI, protein–protein interaction; GO, Gene Ontology; KEGG, Kyoto Encyclopedia of Genes and Genomes.

First, we used the GSVA method to deeply analyze the enrichment of biological signal pathways between each two subtypes. There were significant differences between subtype-I and subtype-II in metabolic pathways such as nitrogen metabolism, arginine and proline metabolism, histidine metabolism, and tyrosine metabolism (Figure 6(a)). In order to further understand the molecular mechanism of m7G-related genes with different CHB inflammatory responses, we further identified the DEGs among the two subtypes and integrated them to obtain 118 DEGs (Figure 6(b)). Then, PPI was used to analyze the interaction between DEGs, and it was found that there was a close association between the DEGs (Figure 6(c)). Next, in order to identify the potential biological functions and pathways of DEGs among subtypes, we performed GO and KEGG analysis. Go enrichment analysis is divided into three parts: biological process (BP), cellular component (CC), and molecular function (MF). The DEGs in BP were mainly enriched in “leukocyte chemotaxis,” “response to chemokine,” and “cellular response to chemokine” chemotaxis pathways. In CC, it is mainly enriched in “spindle” and “kinetochore” parts. In MF, receptor-related molecular functions such as “receptor ligand activity,” “signaling receptor activator activity,” and “cytokine activity” are mainly enriched (Figure 6(d)). KEGG pathway analysis is mainly enriched in “Cytokine–cytokine receptor interaction,” “Viral protein interaction with cytokine and cytokine receptor,” and “Chemokine signaling pathway” (Figure 6(e)). We found that these pathways are related to inflammatory response, viral infection, and immune activation. It is further proved that m7G-related genes play different roles in the progression of CHB disease.

### 3.6. Identification of m7G Gene Associated with CHB Progression

**Figure 7:**
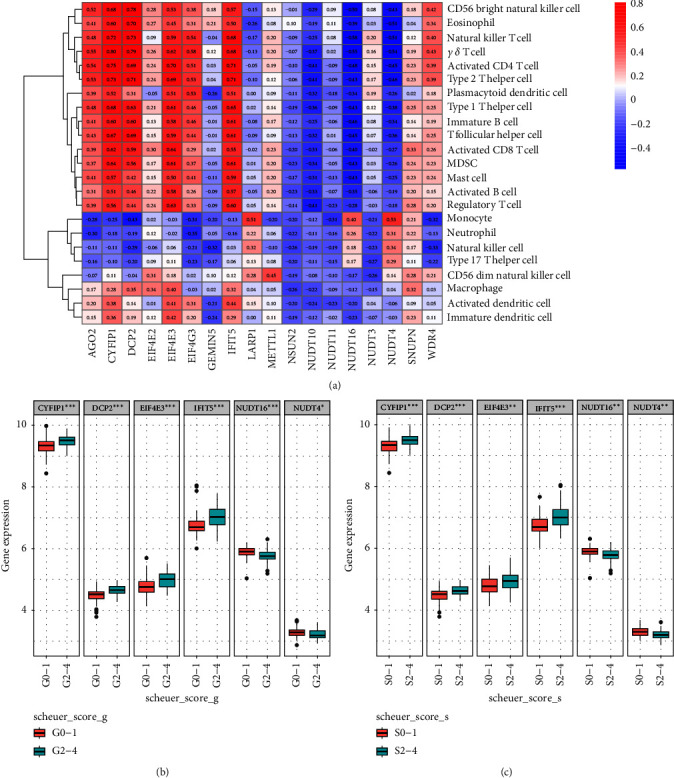
Identification of m7G-related DEGs associated with CHB progression. (a) The heatmap of correlation between m7G-related DEGs and immune cells. (b) The boxplot of expression of m7G-related DEGs in groups with different degrees of inflammation. (c) The boxplot of expression of m7G-related DEGs in groups with different degrees of fibrosis. DEGs, differentially expressed genes; CHB, chronic hepatitis B.  ^*∗*^*p* < 0.05,  ^*∗∗*^*p* < 0.01,  ^*∗∗∗*^*p* < 0.001.

It is known that recurrent immune-mediated liver injury is the main cause of CHB progression to cirrhosis or liver cancer. These results suggest that m7G-related genes are closely related to the immune and inflammatory response of CHB. In order to further clarify which m7G-related DEGs are most closely related to CHB progression, we first analyzed the correlation between m7G DEGs and immune cells. The results as shown in Figure 7(a), *CYFIP1*, *DCP2*, *EIF4E3*, and *IFIT5* were positively correlated with immunocytes such as NKT cells and activated CD8 cells, while *NUDT16* and *NUDT4* were negatively correlated with most immunocytes. In the process of chronic HBV infection, repeated liver inflammation caused by immune cell infiltration is one of the important causes of liver fibrosis [28]. Therefore, we speculate that the upregulation of m7G gene positively related to immune cell infiltration and the downregulation of m7G gene negatively related to immune cell infiltration may be related to the progression of CHB. To prove this, we continued to analyze the patients with HBV related liver fibrosis. In GSE84044 dataset (124 patients with HBV related liver fibrosis), we found that the expressions of *CYFIP1*, *DCP2*, *EIF4E3*, and *IFIT5* were upregulated in patients with moderate to severe inflammation (G2–G4) compared with patients with mild inflammation (G0–G1), while the expressions of *NUDT16* and *NUDT4* were downregulated (Figure 7(b)). Compared with patients without significant liver fibrosis (S0–S1), patients with significant liver fibrosis (S2–S4) also had upregulated expression of *CYFIP1*, *DCP2*, *EIF4E3*, and *IFIT5*, while downregulated expression of *NUDT16* and *NUDT4* (Figure 7(c)). Through the above results, we found that m7G-related genes involved in immune microenvironment regulation play an important role in CHB. This also confirms our hypothesis that m7G-related genes are closely related to the progression of CHB.

## 4. Discussion

So far, various studies have shown that m7G modification is significantly involved in various tumorigenesis and cancer progression, and has become an important regulator in cancer, including HCC [29]. Chronic persistent HBV infection is a complex process involving the interaction between host immune system and virus [30]. Persistent liver inflammation in CHB patients accelerates hepatocyte renewal, leading to liver fibrosis and eventually HCC [31]. To clarify the characteristics of inflammatory response during CHB may provide a new idea for slowing down the progress of the disease. Although the pathological mechanism of CHB has been extensively studied, the role of m7G-related genes in CHB is still unclear. Therefore, it is necessary to explore the relationship between m7G-related genes and liver immune microenvironment in CHB, and to find out the potential predictors of CHB disease progression. Through a series of correlation analysis, this study found potential diagnostic markers in CHB patients and verified them by RT-qPCR. For CHB patients, it is more ideal to find diagnostic markers in peripheral blood samples than liver samples. *LARP1* not only participates in embryogenesis, mitotic spindle pole formation, successful mitochondrial isolation, and cell cycle progression, but also acts as a viral host factor and is upregulated in various tumor tissues, such as cervical cancer and liver cancer [32–34]. The protein encoded by *GEMIN5* is a multifunctional protein, and if its function is disrupted, it may cause defects in extensive mRNA splicing [35]. Previous studies have shown that GEMIN5 can stimulate translation of the survival of motor neuron (SMN) mRNA, and decreased levels of SMN protein can cause muscle diseases such as spinal muscular atrophy [36]. We identified *LARP1* and *GEMIN5* as diagnostic markers for CHB.

Then, we divided CHB patients into two subtypes according to the m7G DEGs between CHB and normal people. We found that the ALT and AST, immune cell infiltration, immune pathway, HLA expression level, and immune checkpoint distribution of the two subtypes were different. Among them, subtype-Ⅰ had the most enriched immune cells and pathways, the heaviest immune response, and differences in metabolic pathways. After analysis, we found that subtype-Ⅰ corresponds to a higher m7Gscore, suggesting that subtype-Ⅰ is closely related to the progression of CHB disease, and further explained that six m7G-related DEGs are involved in the progression of CHB disease. We further found that *CYFIP1*, *DCP2*, *EIF4E3*, and *IFIT5* may be the key genes to promote the progress of CHB, and *NUDT16* and *NUDT4* may protect CHB from further disease progression. In order to prove our conjecture, we confirmed our conclusion in the GSE84044 dataset. m7G-related genes are involved in the progress of CHB. Our study provides new ideas for CHB patients. It is generally believed that HBV infection can induce dysfunction of innate and adaptive immune responses involved in various immune cells [37]. However, CHB with recurrent immune-mediated liver injury eventually leads to cirrhosis and HCC [38]. Moreover, Zhang et al. [39] found that the distribution of immune cells in the liver was different at different stages of HBV infection by single cell sequencing technology. Inflammatory cells release various cytokines and chemokines, which may promote disease progression in CHB patients and even HCC tumorigenesis [40]. We also found that subtype-I has relatively more immune cell infiltration and pathways HBV can induce immunosuppressive cells, such as Treg and myeloid cells, through the immunosuppressive cascade. Excessive immunosuppressive cells can lead to persistent infection of CHB, liver fibrosis, and the progression of HCC [41]. CD8+ T lymphocytes have two sides. On the one hand, CD8+ T cells produce IFN-*γ*, IL-2, TNF-*α*, Granzyme, and perforin can control HBV infection [42]. On the other hand, HBV specific CD8+ T cells can lead to persistent liver inflammation, thus causing the occurrence and development of HCC [43]. It can be seen that CD8+ T cells can promote the disease progression of CHB patients, which is also consistent with our results, that is, subtype-I has a relatively serious inflammatory response. In patients with severe inflammation, the activation of immune cells requires more HLA gene expression. The association of HLA allele variation with disease progression and virus clearance in chronic HBV infection among different ethnic groups is crucial [44]. In the previous work of our research group, it has been confirmed that HLA is closely related to the different degrees of HBV infection [45]. This is consistent with the results of this study that patients with subtype-I with high-inflammatory response and high-immune infiltration have high-HLA gene expression and high-immune checkpoints expression are inhibitory pathways in the immune system and play an immunosuppressive role. Immune checkpoint molecules play an important role in tumor immune escape. There are few studies on the relationship between immune checkpoints and chronic HBV infection. In our research results, most immune checkpoints are most enriched in subtype-I. At present, the effect of immunotherapy on antiviral therapy remains to be determined [46]. Our research provides a reference for the development of immunotherapeutic drugs. In our results, patients with subtype-I had higher HLA and immune checkpoints than those with subtype-II. This also gives us a good indication that patients with subtype-I are more likely to develop liver fibrosis and HCC.

In addition, many studies have shown that multiple pathways such as fatty acid metabolism, amino acid metabolism, and drug metabolism are commonly dysregulated in liver cancer [47–49]. In our study, the two subtypes have different metabolic characteristics, with a low enrichment score of subtype-I metabolic pathways, which further suggests that subtype-I patients have a higher risk of adverse progression.

Therefore, it is reasonable to speculate that m7G-related genes are closely related to the disease progression of CHB patients. m7G gene, which is related to immune cell infiltration in CHB patients, may be involved in disease progression.

Next, we explored the relationship between m7G DEGs and immune microenvironment, and found that m7G DEGs are related to the progress of CHB disease and have stable genes to maintain the current stage of CHB. *CYFIP1*, *DCP2*, *EIF4E3*, and *IFIT5* are positively correlated with immune infiltration in patients with CHB, which may promote the progression of CHB, while *NUDT16* and *NUDT4* are just the opposite. These six genes are involved in the progress of CHB, and consistent results have been obtained in the liver fibrosis population in the GSE84044 dataset, which further confirms our hypothesis. Human genetics research has found that cytoplasmic FMR1 interacting protein 1 (*CYFIP1*) is associated with prominent development and thus participates in a variety of nervous system diseases [50]. In addition, some scholars have proposed that *CYFIP1* may act as a tumor suppressor gene and may have tumor suppressive function [51]. However, recent studies have found that *CYFIP1* is low expressed in acute lymphoblastic leukemia, which may be a potential biomarker of acute lymphoblastic leukemia [52]. Moreover, the regulatory effect of *CYFIP1* on *WASF3*, knockdown in highly invasive cancer cells will lead to inhibition of invasion [53]. It is positively correlated with the promotion of CHB progression in our study. *DCP2* can produce 5′- monophosphate mRNA and m7GDP hydrolysis m7G cap [54]. It is required for mRNA degradation in normal mRNA conversion and nonsense mediated mRNA decay [55]. Eukaryotic translation initiation factor 4E family member 3 (*EIF4E3*) is a series of initiation factors that bind to the 5′ cap of mRNA and regulate proteome and cell phenotype. It is also a prognostic factor for osteosarcoma and can predict the survival time of osteosarcoma [56]. *IFIT5* is involved in tumor metastasis. There is a correlation between *IFIT5* and HPV E6. *IFIT5* may be involved in the malignant transformation of oral squamous cells during disease progression [57]. I*FIT5* enhances antiviral response by enhancing innate immune signaling pathways [58]. Nucleoside diphosphate linked part X (*NUDIX*) motif 16 like 1 (*NUDT16L1*) is associated with adhesion related signal transduction NUDIX hydrolase 16 (*NUDT16*). The hydrolase activity can remove the ADP ribosylation of 53BP1 to regulate its stability and localization at DNA double strand breaks (DSBs) [59]. *NUDT16* gene silencing in HeLa cells is associated with the accumulation of inosine in RNA and the increase of single strand breaks (SSB) in DNA. In addition, this silencing leads to the inhibition of HeLa cell progression [60]. NUDT16 has recently been proved to mediate the selective degradation of Rift Valley fever virus mRNA [61]. Together with *NUDT3*, *NUDT4* forms a subfamily of NUDT hydrolases with widely different relative activities for different adenosine diphosphate and ribosyl diphosphate polyphosphate [62]. It can be seen that m7G-related genes have a broad research foundation and can be used as prediction genes for the next stage of CHB.

These results further show that m7G-related genes are closely related to the immune microenvironment of CHB patients and participate in the disease progression of CHB patients. At present, there are few studies on epigenetics in the field of HBV infection. We took the lead in introducing the m7G mechanism in the process of CHB disease progression, and confirmed that m7G modification participates in the regulation of liver immune microenvironment in the process of CHB progression. The research is pioneering. This study has laid a solid foundation for researchers to carry out m7G-related research in CHB. However, the mechanism between m7G modification and immune microenvironment still needs a lot of basic experiments.

## 5. Conclusion

We found that m7G-related genes were changed in chronic HBV infection, and used SVM-RFE and random forest to screen out diagnostic markers, which were well-proved by RT-qPCR. Next, we found that m7G-related genes are also involved in the regulation of the immune microenvironment. Meanwhile, the m7G gene associated with immune cell infiltration plays an important role in the progression of CHB disease. This will help to further understand the underlying pathogenesis of CHB and provide new ideas for preventing disease progression.

## Data Availability

The authors declare that all the other data supporting the findings of this study are available within the paper and its additional files and from the corresponding author upon reasonable request.

## Supplementary Materials

Supplementary Materials Figure S1: Identification of m7G modified subtypes. Table S1: Clinical characteristics of normal people and patients with CHB. Table S2: Primer sequences.
