# Supplementary material for: Comprehensive Analysis of m7G-Related Genes and Chronic Hepatitis B: Diagnostic Markers, Immune Microenvironment Regulation, Disease Progression
Source: J Immunol Res. 2023 May 10;2023:9471520. doi: 10.1155/2023/9471520 (PMC10191754; doi:10.1155/2023/9471520)
Supplement: Supplementary Materials — Figure S1: Identification of m7G modified subtypes. Table S1: Clinical characteristics of normal people and patients with CHB. Table S2: Primer sequences. [file 9471520.f1.doc]

Supplementary Material

# Supplementary Figure S1 : Identification of m7G modified subtypes.


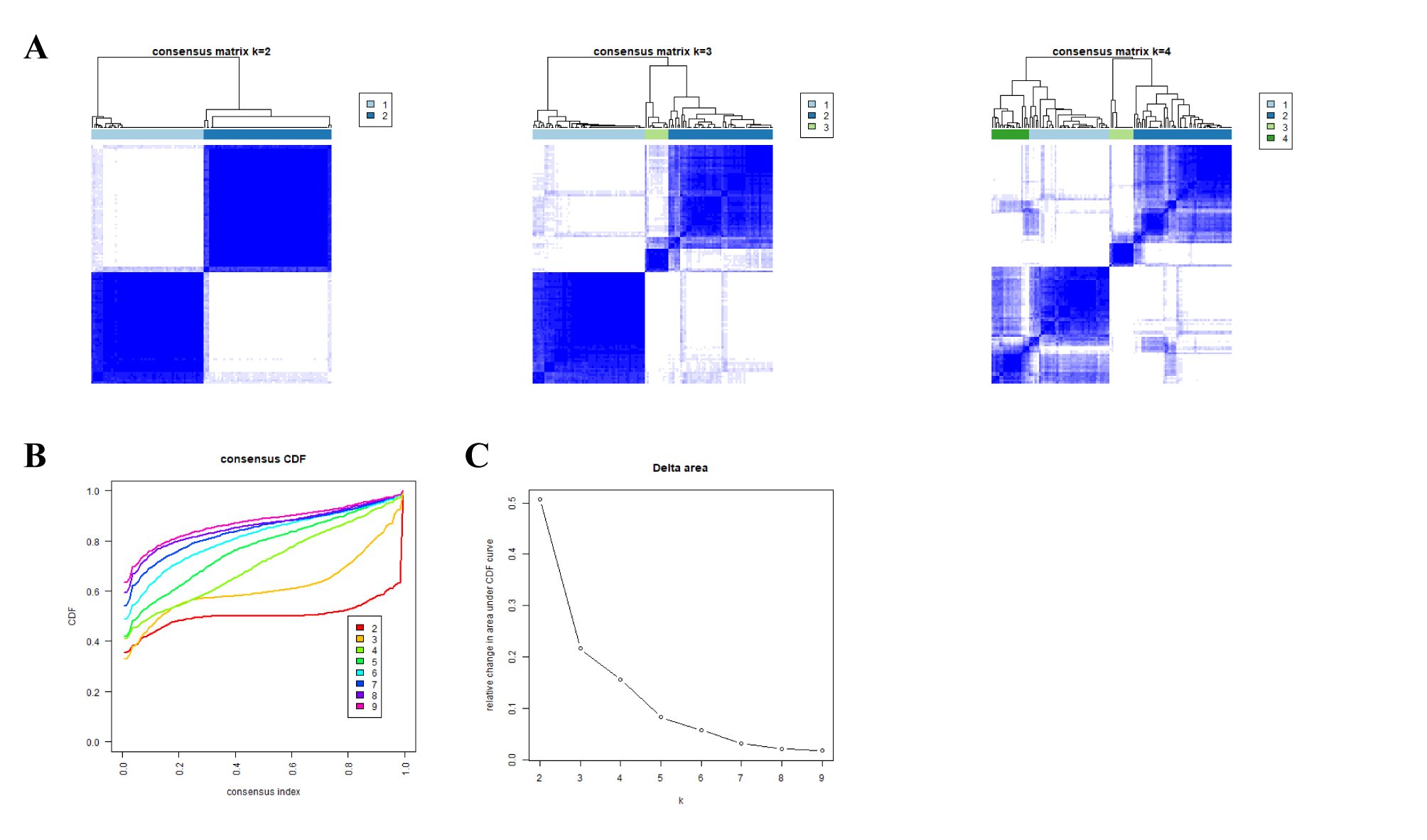


Figure S1: Identification of m7G modified subtypes. (A) Consensus clustering of the m7G-related DEGs. (B) The cumulative distribution functions of consensus matrix for each k value. (C) Relative change in area under CDF curve for k = 2-9. DEGs, differentially expressed genes; CHB, chronic hepatitis B; CDF, cumulative distribution function.

# Supplementary Table S1 : Clinical characteristics of normal people and patients with CHB.

| Variables | Normal (n = 30) | CHB (n = 39) |  |
| --- | --- | --- | --- |
| Age | 32.0 (10.5) | 34.0 (13.0) | p = 0.081 |
| Gender (M/F) | 30 (12/18) | 39 (23/16) | p = 0.118 |
| HBsAg (+/-) | 0/30 | 39/0 |  |
| HBsAb (+/-) | 30/0 | 0/39 |  |
| ALT (H/N) | 0/30 | 23/16 |  |
| AST (H/N) | 0/30 | 14/25 |  |

Note: Data are expressed as median ± quartile range. M/F, Male/female; HBsAg(+/-), HBsAg positive and negative; HBsAb(+/-), HBsAb positive and negative; ALT (H/N), ALT > 50 (male) or ALT > 40 (female), ALT ≤ 50 (male) or ALT ≤ 40 (female); AST (H/N), AST > 40 (male) or AST > 35 (female), AST ≤ 40 (male) or AST ≤ 35 (female).

# Supplementary Table S2 : Primer sequences.

| **Gene** | **Primer nucleotide sequence** |
| --- | --- |
| *LARP1* | Forward: ACACAAGTGGGTTCCATTACAAA |
|  | Reverse: CTCCGCGATTGGCAGGTAT |
| *GEMIN5* | Forward: CCTCCGTCTTCCTTGTCCG |
|  | Reverse: CAGAGACCCTTTCGGTGTGTC |
